# Is the positivity of estrogen receptor or progesterone receptor different between type 1 and type 2 endometrial cancer?

**DOI:** 10.18632/oncotarget.13471

**Published:** 2016-11-19

**Authors:** Fang Shen, Yifei Gao, Jingxin Ding, Qi Chen

**Affiliations:** ^1^ The Hospital of Obstetrics & Gynaecology, Fudan University, China; ^2^ Department of Obstetrics & Gynaecology, The University of Auckland, New Zealand

**Keywords:** endometrial cancer, type 1 and type 2, ER, PR, menopause

## Abstract

Endometrial cancer is a major cancer in women and traditionally divided into type 1 and type 2. It is well known that type 2 endometrial cancer has a poor prognosis. Studies have suggested that estrogen receptor (ER) or progesterone receptor (PR) positive are positively associated with endometrial cancer survive. However whether the positivity of ER or PR is different between cancer types has not been investigated yet. In this retrospective study, the positivity of ER or PR was analysed in 1054 women with primary diagnosed endometrial cancer taking into account cancer types and menopausal status from the largest university teaching women's hospital in China. The positivity of ER or PR (over 90%) was significantly higher in type 1 compared to that in type 2 endometrial cancer (71% or 64%) in both premenopausal and postmenopausal women. There was no different in positivity of ER or PR in type 1 endometrial cancer between premenopausal and postmenopausal women. However, in type 2 endometrial cancer, the positivity of ER or PR in premenopausal women was significantly higher compared to that in postmenopausal women. Our data demonstrate that both ER and PR positivity are significantly higher in type 1 endometrial cancer (92%) compared to type 2 (72% ER positive, 65% PR positive). Menopausal status is not associated with the positivity of ER or PR in type 1 endometrial cancer. Our data may provide some novel insights why Asian women have better outcomes of endometrial cancer which was reported in the literature.

## INTRODUCTION

Endometrial cancer is the major cancer of the female reproductive tract in developed countries and causes more than 10,000 deaths in the United States (American Cancer Society: Cancer Facts and Figures 2016). The exact causes of endometrial cancer are still unclear, however a number of risk factors for developing endometrial cancer such as early menarche and late menopause, nulliparity, obesity, increasing age, hypertension and ethnicity have been identified [1–6]. Most of these risk factors are associated with the changes in levels of sexual hormones during women's lifetime.

Of these sexual hormones, estrogen has a mitogenic effect on endometrial tissue, by stimulating the endometrial glands and stromal cells to grow and proliferate during the menstrual cycle [7, 8]. Studies suggested that unopposed endometrial estrogen exposure, such as estrogen replacement therapy during menopause, is associated with increased risk of developing endometrial cancer [9]. In contrast, it is well-documented that parity is negatively correlated with the incidence of endometrial cancer. Shift in the balance of estrogen and progesterone towards more progesterone (reducing unopposed estrogen) and the reduced number of ovulation during pregnancy have been suggested to contribute this long term protective effect [10].

Endometrial cancer is traditionally divided into estrogen dependent (type 1) and estrogen independent (type 2) [11]. Type 1 endometrial cancer is thought to be caused by excess estrogen following estrogen related pathway. It occurs most commonly before and around the time of menopause and supplementation of estrogen alone (without progesterone) increases the risk for developing type 1 endometrial cancer in women [9]. While type 2 endometrial cancer may not be caused by excess estrogen, because this type cancer usually occurs in older and post-menopausal women [12]. Both estrogen and progesterone exert their effect through intra-and extra-nuclear receptors. The positivity of estrogen receptor (ER) and progesterone receptor (PR) is positively associated with the prognosis of endometrial cancer, including the survival rate and survival time [13, 14]. ER or PR positive in endometrium is also associated with the hormonal treatment in endometrial cancer [15]. It is well-known that type 1 has a better survival rate with treatment, while type 2 has a poorer prognosis with aggressive form of the disease. These studies potentially suggest that the positivity of ER or PR may be different between type 1 and type 2 endometrial cancer. However, other study suggested that the subtypes of endometrial cancer share many common risk factors, and hypothesised that type 2 endometrial cancer may not be completely estrogen-independent [16]. In addition, our recent study (under review) indicated that the levels of sexual hormones including estrogen are in fact not different between type 1 and type 2 endometrial cancer. We also found that only small proportion (less than 20%) of endometrial cancer patients with excess estrogen (under review). This prompted us to question whether there is in fact a difference in ER or PR positivity between type 1 and type 2 endometrial cancer.

Therefore, this study aimed to investigate the positivity of ER or PR in endometrium between type 1 and type 2 endometrial cancer taking into account menopausal status.

## RESULTS

### Clinical characteristics of the study population

The clinical and histological characteristics of study participants are summarised in Table 1. The median age of patients at diagnosis was 54 (range 26–88) years old. Of 1054 patients, 815 (77%) were diagnosed with type 1 endometrial cancer, and 446 (42.3%) patients were diagnosed before menopause. There was no statistical difference in the median age between premenopausal women with type 1 (47 range from 26 to 62 years) and type 2 endometrial cancer (48 range from 26 to 58 years) at diagnosis. There was also no statistical difference in the median age between postmenopausal women with type 1 (58 range from 45 to 88 years) and type 2 endometrial cancer (59 range from 47 to 81 years) at diagnosis.

**Table 1:** Clinical characteristics of the study population

	Women with endometrial cancer (*N* = 1054)
Age at diagnosis (years, median/range)	54 (26–88)
FIGO stage (number, %)	
I II III IV	800 (76%) 122 (12%) 115 (10%) 17 (2%)
Histological type (number, %)	
Endometrioid	835 (79%)
serous	81 (8%)
Mucinous	31 (3%)
Clear cell	33 (3%)
Squamous cell	42(4%)
Mixed	32 (3%)
Premenopause (number, %)	446 (42.3%)
Post-menopause (number, %)	608 (57.7%)
Type 1(number, %)	815 (77%)
Type 2 (number, %)	239 (23%)

### The positivity of estrogen receptor (ER) and progesterone receptor (PR) is higher in type 1 endometrial cancer

Overall, 86.5% of cases were ER positive or 85% of cases were PR positive. We then analysed the positivity of ER or PR between two types of endometrial cancer. In type 1 endometrial cancer, 92.1% or 91.0% of cases were ER or PR positive, whereas in type 2 endometrial cancer, 71.9% or 64.8% of cases were ER or PR positive respectively (Table 2). The positivity of ER or PR in type 1 endometrial cancer was significantly higher than that in type 2 endometrial cancer (*p* = 0.0001 or *p* = 0.0001, respectively, Table 2).

**Table 2:** The expression of estrogen receptor (ER) or progesterone receptor (PR) in endometrial cancer

	Type 1 (*n* = 815)	Type 2 (*n* = 239)	*P* value
ER positive (number, %, lower, upper CL)	751 (92.1%) (90.1%, 93.9%)	172 (71.9%) (65.8%, 77.6%)	*P* = 0.0001
PR positive (number, %, lower, upper CL)	742 (91.0%) (88.8%, 92.9%)	155 (64.8%) (58.4%, 70.9%)	*P* = 0.0001

Menopausal status is one of the risk factors for endometrial cancer, we then compared the positivity of ER or PR in patients before menopause or after menopause according to the cancer types (Table 3). In premenopausal women with type 1 endometrial cancer, the positivity of ER (92.8%) or PR (92.8%) was significantly higher than that in premenopausal women with type 2 endometrial cancer (79.3% or 81.7%) (Table 2, *P* < 0.0001). Similarly, in postmenopausal women with type 1 endometrial cancer, the positivity of ER (91.5%) or PR (90.0%) was significantly higher than that in premenopausal women with type 2 endometrial cancer (53.5% or 58.5%) (Table 2, *P* < 0.0001).

**Table 3:** The expression of estrogen receptor (ER) or progesterone receptor (PR) in endometrial cancer between cancer types according to menopausal status

Premenopause (*n* = 446)	Type 1 (*n* = 364)	Type 2 (*n* = 82)	*P* value
ER positive (number, %)	338 (92.8%)	65 (79.3%)	*P* = 0.0009
PR positive (number, %)	338 (92.8%)	67 (81.7%)	*P* = 0.005
Postmenopause (*n* = 608)	Type 1(*n* = 451)	Type 2 (*n* = 157)	
ER positive (number, %)	413 (91.5%)	84 (53.5%)	*P* < 0.0001
PR positive (number, %)	406 (90.0%)	92 (58.5%)	*P* < 0.0001

### The positivity of estrogen receptor (ER) or progesterone receptor (PR) is associated with menopausal status in type 2 not in type 1 endometrial cancer

We further investigated whether the positivity of ER or PR is associated with menopausal status taking into account cancer types. In premenopausal women with type 1 endometrial cancer (*n* = 364), 338 cases (92.8%) were ER or PR positive. In postmenopausal women with type 1 endometrial cancer (*n* = 451), 413 cases (91.5%) or 406 cases (90.0%) were ER or PR positive. There was no difference in the positivity of ER or PR in type 1 endometrial cancer between premenopausal and postmenopausal patients (Table 4, *p* = 0.587 or *p* = 0.191). However, in type 2 endometrial cancer, 65 (79.5%) or 67 (81.7%) premenopausal patients were ER or PR positive, which was significantly higher than that in postmenopausal patients (53.5% were ER positive or 58.5% were PR positive) (Table 4, *p* = 0.0001 or *p* = 0.0004).

**Table 4:** The expression of estrogen receptor (ER) or progesterone receptor (PR) in endometrial cancer between premenopause and postmenopause

Type 1 (*n* = 815)	Premenopause (*n* = 364)	Postmenopause (*n* = 451)	*P* value
ER positive (number, %)	338 (92.8%)	413 (91.5%)	*P* = 0.587
PR positive (number, %)	338 (92.8%)	406 (90.0%)	*P* = 0.191
Type 2 (*n* = 239)	Premenopause (*n* = 82)	Postmenopause (*n* = 157)	
ER positive (number, %)	65 (79.3%)	84 (53.5%)	*P* = 0.0001
PR positive (number, %)	67 (81.7%)	92 (58.5%)	*p* = 0.0004

## DISCUSSION

The positivity of estrogen receptor (ER) or progesterone receptor (PR) has been shown to be positively associated with the prognosis of endometrial cancer [17, 18]. Endometrial cancer with early stage or lower grade usually retains the expression of both receptors, but endometrial cancer with advanced stage or poor differentiation often lack one or both of these receptors. In this study, we found overall 85% cases of endometrial cancer were ER or PR positive. In comparison to other studies showing around 60–75% positivity of ER or PR in Caucasians [19, 20], the positivity of ER or PR in this study is higher. Ethnicity is one of the risk factors for developing endometrial cancer and black women have a higher incidence of endometrial cancer. However, a study suggested Asian women with endometrial cancer have improved outcomes and better survival rate compared to non-Asian women [21]. This may be associated with higher positivity of ER or PR in Chinese (Asian) population with endometrial cancer.

It is well-known that type 2 endometrial cancer has a poor prognosis including survival rate and time compared to type 1 endometrial cancer. However, to date most studies investigated the association between the positivity of ER or PR and prognosis without taking into account cancer types and there is current no study investigating the positivity of ER or PR between two types of endometrial cancer. In this study, our data show that the positivity of ER or PR in type 1endometrial cancer is significantly higher than that in type 2 endometrial cancer in both premenopausal and postmenopausal women. Our data suggest that the higher positivity of ER or PR in type 1 endometrial cancer is not associated with the menopausal status. However, in this study we also found that the positivity of ER or PR in type 2 endometrial cancer was 72% or 65%, suggesting the majority of endometrial cancer were ER or PR positive regardless of cancer types in Chinese population. We do not know the exact reason why the majority type 2 endometrial cancer were ER or PR positive, but ethnicity may be one of the reasons, because study recently reported that Asian women have improved outcomes and better survival rate in endometrial cancer compared to non-Asian women [21].

The incidence of endometrial cancer is an ethnicity and geographical region dependent [22, 23]. Endometrial cancer commonly occurs in postmenopausal women in Caucasians. However, our recent study found that endometrial cancer also frequently occurs in Chinese women before menopause [24]. Therefore we investigated the positivity of ER or PR in women with endometrial cancer taking into account menopausal status. In our current study we found that the positivity of ER or PR was not different between premenopausal and postmenopausal women with type 1 endometrial cancer. However, in type 2 endometrial cancer, the positivity of ER or PR in premenopausal women is significantly higher than that in postmenopausal women. Our data suggest that postmenopausal women with type 2 endometrial cancer may have poorer prognosis compared to premenopausal women with type 2 endometrial cancer. But this needs to be investigated in future study.

Studies suggested that ER positive has no significant prognostic relevance, but PR positive has [20]. Endometrial cancer with higher positivity of PR has a good prognosis compared to that with lower positivity of PR. The effects of progesterone are mediated through interaction with PR by inhibition of endometrial cancer cell proliferation and invasion, and increased sensitivity to apoptotic stimuli [25, 26]. PR is normally negative in advanced endometrial cancer, such as grade 3 endometrial cancer [27]. Studies reported that 93% of women with PR positive survived to three years, compared with 36% of women with PR negative [28, 29]. However, in our current study we found there was no difference between the positivity of ER and the positivity of PR in both type 1 and type 2 endometrial cancer regardless of the menopausal status.

In conclusion, to our knowledge, this is the first study reporting the positivity of ER or PR between type 1 and type 2 endometrial cancer with large sample size. Our data demonstrate that the positivity of ER or PR in type 1 endometrial cancer is significantly higher than that in type 2 endometrial cancer regardless of menopausal status. However, the majority of type 2 endometrial cancer is still ER or PR positive in Chinese population. In addition, we found that the positivity of ER or PR in type 2 endometrial cancer in premenopausal women is significantly higher than that in postmenopausal women. Our data suggest that menopausal status may not be associated with prognosis in type 1 endometrial cancer but it may be associated with prognosis in type 2 endometrial cancer. Our data may also provide some novel insights why Asian women have better outcomes of endometrial cancer which was reported in the literature.

## MATERIALS AND METHODS

This study was approved by the Ethics Committee of The Hospital of Obstetrics & Gynaecology, Fudan University of China.

### Study participants

The retrospective data were collected from the electronic based medical records of patients from January 2011 to December 2014 from The Hospital of Obstetrics & Gynaecology, Fudan University, China which serves a diverse urban and rural population in China. In this study, data from 1054 women with a primary diagnosis of endometrial cancer were included. Clinical characteristics included age at diagnosis, self-reported age at menopause, parity and pathological findings of endometrial cancer.

The classification of type 1 and type 2 endometrial cancer was determined by pathological examination of biopsies, including cancer histologic subtypes and grades. We classified endometrioid and adenosquamous carcinoma with grade 1 and 2 as type 1 endometrial cancer. Clear-cell, serous, mucinous carcinoma and grade 3 endometrioid carcinoma were classified as type 2 endometrial cancers, according to the classification of the International Federation of Gynaecology and Obstetrics (FIGO) (Table 1).

Endometrial cancer was diagnosed first by a physical examination and then endometrial biopsy. The endometrial tissue was examined histologically for characteristics of cancer including types of cancer.

### Immunohistochemistry

Estrogen or progesterone receptors were examined by immunohistochemical methods on paraffin-embedded tissue. The expression of ER and PR in endometrial tissue (*n* = 179) was measured by immunohistochemistry on paraffin-embedded sections. Briefly, antigen retrieval was performed by treatment with citric acid (pH 6.0) for 20 minutes. Non-specific antibody binding was blocked by incubating with 10% fetal calf serum for 20 minutes. Mouse anti-human ER (1:200) or PR monoclonal antibody (1:000, Dako, Shanghai, China) were added for 1 hour at room temperature. Sections were then washed with phosphate-buffered saline (PBS) and incubated with biotinylated anti-mouse IgG (Dako, Shanghai, China) for 30 minutes, and after washing sections were then incubated with streptavidin-conjugated horseradish peroxidase (Dako, Shanghai, China) for 30 minutes. The antigen–antibody complexes were visualised using 3,3-Diaminobenzidine (DAB) and counterstained with haematoxylin. The cut-off point of 1% positive cells was considered as ER or PR positive.

### Statistical analysis

The statistical difference in positivity of estrogen or progesterone receptor in patients with type 1 or type 2 endometrial cancer in premenopausal and postmenopausal women was assessed by the Fisher Exact test using the Prism software package (GraphPad Software Inc, San Diego, CA, USA) with *p* < 0.05 being considered as statistically significant.
